# CSF ADA Determination in Early Diagnosis of Tuberculous Meningitis in HIV-Infected Patients

**DOI:** 10.1155/2016/5820823

**Published:** 2016-04-06

**Authors:** Gopal Chandra Ghosh, Brijesh Sharma, B. B. Gupta

**Affiliations:** ^1^Hospital Annexe, Christian Medical College, Hospital Campus, Room No. 310, Vellore 632004, India; ^2^PGIMER and Dr. RML Hospital, Main Block, New Delhi 100001, India

## Abstract

Tuberculous and Cryptococcal meningitis are common in HIV patients. A highly specific and sensitive rapid test for diagnosis of Tuberculous meningitis especially in setting of HIV is not available in developing countries where the burden of disease is high. We measured ADA (adenosine deaminase) levels using spectrophotometric method in the CSF of HIV patients with meningitis to differentiate Tuberculous meningitis from meningitis due to other causes. Kruskal-Wallis test was used to compare ADA values between tuberculous meningitis (TBM) and nontuberculous (non-TB) meningitis patients and a receiver-operating characteristic (ROC) analysis curve was drawn from these values. Levels of ADA in the CSF of patients with TBM were significantly higher than those in patients with meningitis due to other causes. CSF ADA level determination with a cut-off value of 6 IU/L was found to be highly specific and fairly sensitive test for the diagnosis of TBM in HIV positive patients.

## 1. Introduction

Tuberculous meningitis (TBM) is an endemic disease in developing countries [1], more so in patients with a low socioeconomic status. TBM remains a major global health problem [2, 3].

Five lakh patients die of tuberculosis every year in India [4]. The outcome is the worst in multidrug resistant tuberculosis [5]. Studies from India have reported HIV seropositivity rates in patients with tuberculosis to be between 0.4 and 20.1% [6]. On the other hand, 50% of HIV-infected patients in India are coinfected with* M. tuberculosis* and approximately 200,000 of these coinfected persons will develop active tuberculosis each year in association with HIV infection [7]. Mortality is significantly higher in HIV-infected patients with tuberculous meningitis (63.3%) as compared to HIV negative patients (17.5%) [8]. Methods of diagnosis of TBM have been found to have low sensitivity and specificity [9].

Newer methods for diagnosing tuberculosis are based on phenotypic and genotypic techniques. For the detection of acid fast bacilli (AFB) in a smear, light microscopy is a common, rapid, and specific method and is used worldwide with a detection rate of 30–40% [10]. Culture using Lowenstein-Jensen (L-J) medium has sensitivity higher than microscopy but needs several weeks of incubation. A number of genotypic assays based on nucleic acid amplification have been designed including Gen-Probe amplified* Mycobacterium tuberculosis* direct test, Roche Amplicor MTB test, Cobas Amplicor test, Abbott LCx test, and the BD-Probe Tec (strand displacement amplification) test [11–15]. However, high cost precludes their widespread use in developing countries. A simple and cost-effective test for the diagnosis of tuberculous meningitis in HIV positive patients would help to make diagnosis easier.

The definitive criterion for the diagnosis of tuberculous meningitis is demonstration of* M*.* tuberculosis* in CSF, by either direct ZN stained smears or biological culture. However, the sensitivity of CSF ZN staining is 2–87%, and CSF culture is positive for* M*.* tuberculosis* in 25–75% of cases [16–22]. Also, routine CSF laboratory findings may not help to establish etiology of meningitis. Thus, CSF ADA levels measurement may be a rapid and important test to differentiate TBM from other causes of meningitis.

## 2. Materials and Methods

HIV positive patients with meningitis admitted in our department were included in the study. Informed consent was taken from all patients. A detailed history and a clinical examination were performed in all patients. Noncontrast CT scan of the brain was performed followed by lumber puncture under aseptic conditions. A case of tuberculous meningitis was defined as

(1) *Mycobacterium tuberculosis* detected in CSF by Ziehl-Neelsen staining or polymerase chain reaction for* Mycobacterium tuberculosis* or
(2) tuberculosis at another anatomical site with characteristic clinical and CSF findings or
(3) characteristic CSF findings with improvement after starting antituberculous therapy.

**Figure 1:**
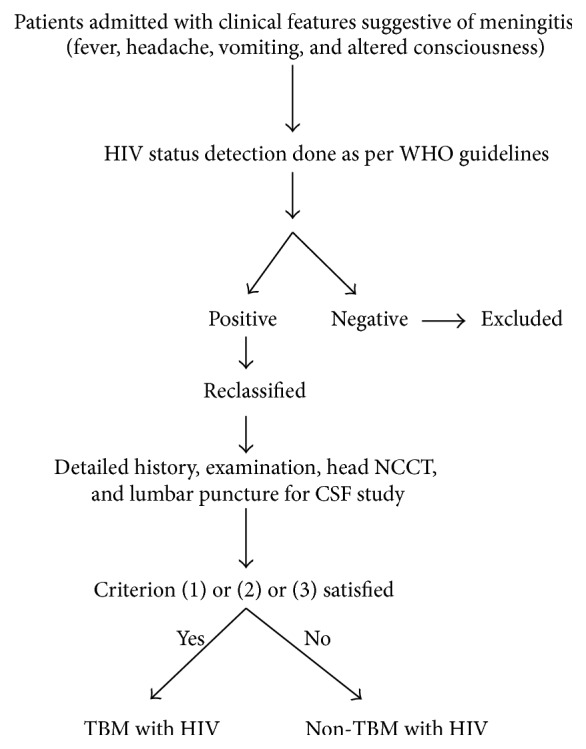
Flow chart showing recruitment of patients.

In order to satisfy criterion (2) or (3), India ink staining, latex agglutination test for* Cryptococcus*, fungal culture, VDRL test, Gram staining, bacterial culture, and latex agglutination test for* Streptococcus pneumoniae, Neisseria meningitidis, Haemophilus influenzae,* and* Listeria monocytogenes* in the CSF must be negative. The recruitment and procedure are shown in Figure 1.

ADA levels were estimated in all samples of CSF using the GALANTI and GIUSTI methods.

### 2.1. Statistical Analysis

ADA levels between patients with tuberculous meningitis and nontuberculous meningitis were compared using the Kruskal-Wallis test. ROC curve analysis was performed to determine the cut-off value for ADA in order to differentiate TBM from non-TBM groups. Statistical analysis was performed using SPSS version 16. A *P* value < 0.05 was considered significant.

## 3. Results

We included 40 HIV positive patients with meningitis in our study; of these, 16 were diagnosed as having tuberculous meningitis (TBM) and 24 patients had nontuberculous meningitis (non-TBM). The mean ± SD age of the patients was 35 ± 10 years with the youngest patient being 16 years old and the oldest 65 years old. There were 32 men and 8 women.

**Figure 2:**
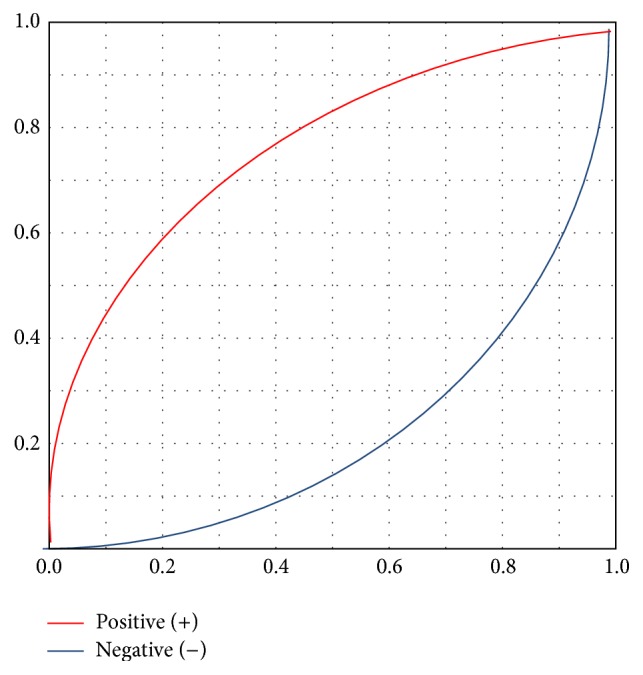
Posttest probability graph for the ADA cut-off value of 6 IU/L in CSF.

**Figure 3:**
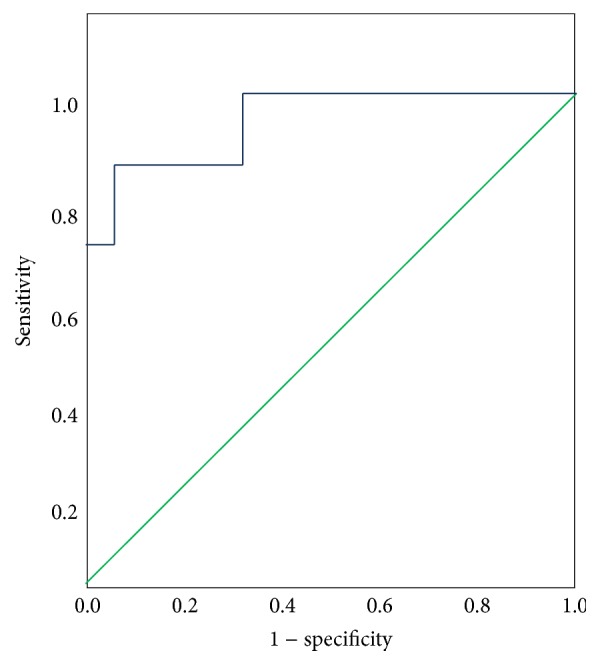
The receiver-operating characteristic (ROC) analysis curve for ADA in tuberculous meningitis and nontuberculous meningitis. Area under curve (AUC) = .958.

Mean ± SD ADA levels in patients with TBM were 18.1 IU/L ± 19.176 IU/L and mean ADA levels in non-TBM patients were 2.2 IU/L ± 1.8 IU/L (*P* < 0.001). Posttest probability was high at ADA value of 6 IU/L and receiver-operating characteristic (ROC) curves analysis of ADA levels in TBM and non-TBM groups revealed area under curve (AUC) of .958 (Figures 2 and 3). A cut-off value of 6 IU/L for CSF ADA activity had sensitivity of 75%, specificity of 95.8%, a positive predictive value of 92.3%, and a negative predictive value of 85.2% for the diagnosis of TBM.

## 4. Discussion and Conclusion

Determination of ADA levels in CSF is a rapid test to distinguish TBM from non-TB meningitis especially in immunocompetent hosts. Its sensitivity ranges from 75% to 94% and specificity from 86% to 97% [23–27]. But the role of CSF ADA level determination is still controversial in meningitis patients with HIV as very few studies are available in the literature. A study by Corral et al. [28] found that CSF ADA level cut-off point of 8.5 IU/L for the diagnosis of tuberculous meningitis had 57% sensitivity and 87% specificity; however, another study by Machado et al. [29] found that elevated CSF ADA levels are nonspecific for diagnosis of TBM in HIV positive patients.

We found that a CSF ADA cut-off value of 5 IU/L has sensitivity of 87.5% and specificity of 87.5%. On the other hand, a cut-off value of 6 IU/L has sensitivity of 75% and specificity of 96.8% for the diagnosis of TBM in HIV positive patients. High specificity makes this a useful investigation for confirming a diagnosis of TBM in HIV positive patients.

A study by Corral et al. [28] revealed that false-positive results were found in patients with neurological CMV disease and cryptococcal, lymphomatous, and probable candidal meningitis. Patients with HIV associated neurological disorders showed higher ADA levels than patients without neurological disease, but the values were usually within normal ranges. Thus, neither HIV infection per se nor HIV infection of central nervous system causes elevation of ADA levels in cerebrospinal fluid.

In our study a cut-off value of 5 has sensitivity of 87.5% for diagnosis of tuberculous meningitis but if the cut-off increases to 6 it further increases the specificity to 96.8%, lowering the sensitivity to 75%. But if we consider the cost-effectiveness of the test, disease burden in developing countries, and need of a rapid accurate diagnostic test, then cut-off value of 6 IU/L has very high specificity and a fair percentage of sensitivity for diagnosis of tuberculous meningitis in HIV positive patients.

Our study result is differing from the study done by Machado et al. This can be because, unlike Machado et al., in our study, none of the patients had candidal meningitis, CMV neurological disease, or neurobrucellosis. On the other hand, our study showed that cryptococcal meningitis is the commonest type of meningitis in HIV positive patients (19 patients). Though the number of cases in our study was small, it demonstrated a statistically significant difference in cerebrospinal fluid ADA levels between tuberculous meningitis and cryptococcal meningitis cases. We need larger studies to further substantiate the role of CSF ADA determination in HIV associated TBM.

## Abbreviations

WHO: World Health Organisation
NCCT: Noncontrast computed tomography scan
CSF: Cerebrospinal fluid
TBM: Tuberculous meningitis.
